# Acupuncture for Depression: Patterns of Diagnosis and Treatment within a Randomised Controlled Trial

**DOI:** 10.1155/2013/286048

**Published:** 2013-06-27

**Authors:** H. MacPherson, B. Elliot, A. Hopton, H. Lansdown, S. Richmond

**Affiliations:** ^1^Department of Health Sciences, University of York, Heslington, York YO10 5DD, UK; ^2^Northern College of Acupuncture, 61 Micklegate, York YO1 6LJ, UK

## Abstract

*Background*. In a large randomised controlled trial of acupuncture, counselling, or usual care for depression, we document the acupuncture intervention and explore the relationship between traditional acupuncture diagnosis and outcome. *Methods*. Patients who were continuing to experience depression were recruited from primary care to the ACUDep trial (*n* = 755). Practitioners documented for each patient the traditional Chinese medicine diagnosis, the points needled, and additional components of the treatment, such as lifestyle advice, as recommended by the STRICTA guidelines. *Results*. Over an 18-month period, 23 acupuncturists delivered 2741 treatments to 266 patients, an average of 10 sessions per patient. The primary and secondary zang fu syndromes were identified for 99% of patients. When combining primary and secondary diagnoses, there was a predominant Liver Qi Stagnation cluster (66% of patients) and a Spleen Deficiency cluster (34%). Practitioners sought de qi responses 96% of the time. Lifestyle advice was given to 66% of patients, most commonly dietary. When comparing patient outcomes, no significant differences were found between the two main syndrome clusters. *Conclusion*. In this large-scale trial, our documentation of diagnosis and treatment provides a useful snapshot of common patterns that patients present with when continuing to experience depression after consulting in primary care.

## 1. Introduction

Trials of acupuncture have often been criticised for providing insufficient details of the intervention [1]. It was in this context that the STandards for Reporting Interventions in Clinical Trials of Acupuncture (STRICTA) recommendations were first developed and then subsequently revised as part of the Consolidated Standards of Reporting Trials (CONSORT) family of reporting guidelines in 2010 [2]. Adequate reporting of interventions in randomised controlled trials is necessary to facilitate interpretation of the results of the trial and make judgements regarding transferability and generalisability. Though the STRICTA recommendations provide a framework for better reporting of interventions in clinical trials of acupuncture, a survey of authors discovered that journal editors, when reviewing manuscripts of acupuncture trials, sometimes required cuts to the reporting on the intervention in order to keep to a minimum article word length [3]. Given the problem that some journals limit the word length of articles, a solution is to publish a separate article focused on the intervention itself, a solution that is also encouraged by the STRICTA/CONSORT collaboration [2].

Acupuncture when practiced according to traditional principles and delivered in routine care is often described as a complex intervention [4]. The multiple components of acupuncture that have been identified can be clustered in three domains [5], namely, needling-related components, nonspecific components such as time and attention with an empathetic practitioner, and acupuncture-related components beyond needling which include the explanations and lifestyle advice that are specific to acupuncture theory [6, 7]. Documenting the specific components of acupuncture within a clinical trial is challenging, especially within a pragmatic clinical trial where practitioners are encouraged to practice as closely as possible to how they would practice routinely [8].

Accordingly, in this paper we report in depth on the acupuncture intervention that was provided within a large trial of acupuncture and counselling for depression, including the methods used to develop the treatment protocol, the additional processes required given the severity of the depression at baseline, and the relationship between diagnosis and outcome.

## 2. Methods

In a three-arm pragmatic randomised controlled trial, we randomised patients to acupuncture, counseling, and usual care as interventions in the proportions of 2 : 2 : 1, respectively. This trial was designed to evaluate whether referral of people who had consulted in primary care with depression, yet continued to be depressed, and were seeking alternatives would benefit or not from a referral to acupuncturists who practised in the style of traditional Chinese medicine. This trial design is an open one, with no patient blinding, because our research question was not seeking to separate out specific from nonspecific effects but rather to evaluate overall benefit. 

Eligible participants were 18 or over, had consulted their general practitioner with depression within the past 5 years, and were continuing to experience “moderate” to “severe” depression with a score of 20 or above on the Beck Depression Inventory (BDI-II) at baseline [9]. 

Ethical approval was obtained in October 2009 (NHS ref. no: 09/H1311/75, trial registration ISRCTN63787732). (The protocol has been published [10], and the clinical and cost-effectiveness results are reported separately.)

The acupuncture treatment was based on the principles of traditional Chinese medicine and provided by members of the British Acupuncture Council (BAcC) with at least three-year postqualification experience. Members of the BAcC have completed a three-year (or equivalent) degree level qualification in acupuncture. We documented the gender of participating acupuncturists, the duration of their initial training in acupuncture, and their postqualification years in practice.

An acupuncture treatment protocol was established prior to the trial, based on a series of pretrial activities. These activities were developed in a way that was consistent with the Medical Research Council's guidelines on developing and evaluating complex interventions [11]. Firstly we conducted a consensus-based study eliciting from a representative group of acupuncture practitioners what might be considered good practice in the treatment for depression [12]. A nominal group technique was used and the principal components and theoretical frameworks underlying an acupuncture intervention for depression were generated from the literature, from other experts, and from those participating in the consensus process. Out of this process we designed a draft treatment protocol that provided some standardisation on the basis of the theoretical frameworks used but allowed flexibility so that practitioners could take into account actual patient variation. In determining this, we followed the method put forward by Hawe et al. that standardisation of complex interventions could be on the basis of function and process, provided they were guided by theory [13]. This allowed the form of the intervention to be tailored to each patient at each session. Secondly, we conducted a pilot randomised controlled trial [14], one of the aims of which was to test the working draft of the acupuncture treatment protocol, which was set out in a manual, with the aim being that we would learn how to improve it for the full-scale trial. In parallel we tested a logbook, to be completed by participating acupuncturists for each patient. Finally we conducted a series of induction sessions for acupuncturists recruited to the trial, and we consulted them on various aspects of the protocol. We then finalised the manual that set out the treatment parameters for participating in the trial and the logbook to be used for reporting actual treatments provided. These documents provided a standard framework for the acupuncturists treating people with depression within the trial. Treatment Guidelines set out a list of 20 processes intended to guide the treatment of trial patients.

The following is a list of processes that the acupuncturists agreed to follow as a guide to their treatment of patients in the trial, while treating patients as closely as possible to how they normally would.

1. Take each patient's history including past medical history, recent medical history, medication usage, and detailed summary of his/her depression and related symptoms.
2. Examine each patient using the TCM diagnostic assessments you find appropriate (e.g., palpation, tongue, and pulse).
3. Make a diagnosis based on the TCM pattern of differentiation you find appropriate (e.g., *zang fu*, eight principles, and four levels).
4. Select a treatment principle based on TCM theory (e.g., tonify Yin and soothe the Liver). Note that the treatment principle may vary for each treatment and that these guidelines allow for such variation.
5. Design a treatment strategy that incorporates the treatment principle and that aims to accomplish the treatment objective.
6. Select acupuncture points and adjunctive therapies (see below) that are suitable for each patient's condition and treatment strategy. These may be modified at subsequent treatment sessions as appropriate.
7. Additional therapies that may be used are restricted to the following:
  - cupping,
  - ear seeds,
  - electrical stimulation,
  - moxa or moxa essence,
  - heat lamp,
  - acupressure (not more than 10 minutes).
8. Note that the use of herbs and magnets is not permitted in the trial.
9. Use acupuncture needles that are of the appropriate length and gauge for the points selected and patient being treated.
10. Insert acupuncture needles using your chosen method of insertion and to the depth you determine is appropriate for the point location and patient.
11. Use needling techniques which correlate with the treatment principle and which elicit the desired response (e.g., *de qi*).
12. Retain the needles for an appropriate period of time according to the point location and the patient's condition.
13. If appropriate, apply the relevant additional therapies (see point 6, above) that supplement each patient's treatment strategy according to the appropriate theory for that therapy.
14. Provide appropriate lifestyle advice according to each patient's needs and your clinical judgement, such that the only advice given is based on acupuncture theory. Note that advice that is not related to acupuncture theory is proscribed.
15. Answer any questions the patients may have about what to expect from acupuncture treatment, their diagnoses, and their prognoses. Where appropriate, explain to each patient about his/her condition from a TCM point of view.
16. Discuss the treatment plan with each patient being sure to include information about the number and frequency of treatments based on his/her needs. Keep in mind that only 12 treatments are funded by the trial and that it is expected that all treatments are completed within 18 weeks of the first treatment. If the patient does not attend a session, please follow the procedures.
17. Discuss and agree on reasonable expectations with each patient regarding his/her acupuncture treatment. Make the patient aware of possible reactions to treatment and that symptoms may worsen initially. Adverse events need to be reported.
18. Record and interpret patients' adverse reactions to acupuncture treatment.
19. Refer patients to their general practitioner or appropriate health care professional if you become concerned about worsening of symptoms or the appearance of new symptoms, or if patients have questions about their prescribed medication.
20. In the event that you become concerned about the safety of your patient, or someone else close to them whom you feel may be at risk, then you must follow the procedures related to suicide intent.
21. Record the points used, adjunctive therapies, and lifestyle advice in the Acupuncture Treatment Log for each patient at each session.
22. At the last session complete the last page of the log which requests information on patient outcome, as well as details of the theoretical framework(s) that you would define as central to your course of treatment for this patient.

To ensure that acupuncturists had the necessary awareness of the level of risk of suicide or self-harm, we asked the acupuncturists to complete the Patient Health Questionnaire (PHQ-9) [15] with participants on their first session. Based on scoring of the final question of the PHQ-9, patients were considered at risk if they had thoughts that they would be better off dead or hurting themselves in some way “nearly every day.” For these cases, the practitioners followed a risk of suicide/self-harm algorithm. The first step was to check with the patient that they had discussed risk with their general practitioner, and if not then they asked their patient to do so. If the patient was unwilling to contact their general practitioner, then as a last resort we requested practitioners to follow the BAcC Code of Professional Conduct, which states that, “disclosures without consent may be necessary in the public interest if your duty to society overrides the duty to the patient.” If necessary the acupuncturist could consult the research team. One of the coauthors (HL) had a supervisory role throughout the trial, supporting the acupuncture practitioners, especially where risks of suicide and self-harm were concerned. Another coauthor (SR) managed the risks of suicide and self-harm from enrolment to study completion based on information gathered from patients and practitioners (including baseline and follow-up PHQ-9 responses) throughout the trial. 

The acupuncturists provided up to 12 sessions for each patient usually weekly, with scope for more frequent sessions early in the course of treatment and less frequent in the later stages. Practitioners recorded the overall length of time for each session, including all consulting time. The primary theoretical frameworks of traditional Chinese medicine, which were prespecified within the trial documentation, were *zang fu* syndromes, eight extra vessels, six divisions, five elements, Qi, blood, body fluids, eight principles, four levels, and pathogenic Factors [16]. Where *zang fu* syndromes were utilised, we asked practitioners to identify both the primary and the secondary syndromes.

The choice of acupuncture points was determined by each acupuncturist at each session, on the basis that it was driven by traditional acupuncture theory and designed to alleviate the symptom complex defined by the acupuncture diagnosis. Acupuncturists also decided on a patient-by-patient and session-by-session basis the depth of needle insertion, the local response elicited (e.g., the *de qi* sensation), and the provision of specific needle stimulation methods. We collected data from each acupuncturist on these aspects of their treatment as an average over all the patients whom they treated in the trial, as well as information on the material of the acupuncture needles they used and their diameter and length.

Therapies auxiliary to acupuncture are often provided in conjunction with usual acupuncture practice [7]. Within the trial protocol the following were allowed: moxibustion (also known as moxa), electroacupuncture, ear seeds, cupping, brief acupressure massage, and heat lamp. Herbal medicines and magnets were proscribed. Lifestyle advice related to the acupuncture diagnosis was allowed, on the basis that it is commonly offered in the routine practice of acupuncture in the UK [6] as well as more widely in Europe and China [7]. Options included advice about diet, exercise, relaxation, rest, and work. Where such advice was given, the practitioner was asked to explain the rationale based on the relevant acupuncture theory and/or diagnosis. Advice not directly linked to acupuncture theory was proscribed. There was scope to override the treatment protocol, for example, by giving non-acupuncture-related advice, provided the rationale was clearly reported by the acupuncturist in the participant's logbook. 

Patients receiving acupuncture within the trial had access to their usual care, whether for depression or for any other condition. Usual care might consist of consulting their general practitioner or practice nurse, making hospital visits, and taking prescribed or over-the-counter medication. Private health care was also considered part of usual care. Participants documented all aspects of usual care over the 12 months of their time within the trial. These data will be reported with the main trial findings.

SPSS 20 was used for the whole statistical data analysis. Data within groups were analysed using descriptive statistics. Relationships between the acupuncture diagnosis and baseline variables were evaluated using cross tabulation and chi-squared test. Differences in change between groups at three months were assessed using analysis of covariance (ANCOVA) testing. A probability of *P* < 0.05 was considered to be statistically significant.

## 3. Results

In total, 755 participants were recruited and randomised within the ACUDep trial between November 2009 and April 2011. Of these, 302 were allocated to acupuncture. The research team were able to contact only 285 patients within the acupuncture group to organise their first appointment for treatment and 266 received at least one treatment.  Table 1 presents the characteristics of the patients at baseline, based on the 266 patients, and the characteristics of treatment and the practitioners.

Patients in the acupuncture group received a total of 2741 treatments. Based on the 302 patients randomised, this equates to a mean take-up of 8 sessions of the 12 allocated per patient. However, with only 266 taking up the offer of acupuncture, this equates to a mean of 10 sessions attended. 

Practitioners were asked to record all of the theoretical frameworks of traditional Chinese medicine that guided their diagnosis and treatment for each patient (Table 2). The predominant theoretical framework, which was used on 99% of patients, was that based on the *zang fu* syndromes. 

For each patient with a *zang fu* diagnosis, practitioners were asked to record the primary *zang fu* syndrome. In total 38 syndromes were recorded (Table 3). On extracting these data from logbooks, 7 were not considered to be “*zang fu*” syndromes. The remaining syndromes were grouped into the 7 categories. The most common cluster of primary syndromes reported was those related to Liver Qi Stagnation (41%), followed by Spleen Deficiency (16%) and Heart Deficiency (8%). 

Secondary *zang fu* syndromes were also reported by practitioners. These presented a similar picture to the primary syndromes, with Liver Qi Stagnation providing the most common secondary diagnosis (25%), followed by Spleen Deficiency (18%) and Kidney Deficiency (10%). When accounting for both primary and secondary patterns, 66% of participants were diagnosed with some degree of Liver Qi Stagnation, 34% with some degree of Spleen Deficiency, and 18% with Kidney Deficiency. 

Practitioners reported their prescription of acupuncture points for each patient at each session. In total, 246 individual acupuncture points were used across all patients in all sessions. A total of 21,055 point locations were needled, with the 20 most common being listed in Table 4.

The majority of practitioners sought a *de qi* response (256/266; 96%) and used varying methods of stimulation for each patient. The methods used were Tonifying (180/266; 68%), Reducing (114/266; 43%) and Even methods (146/266; 55%). 

The treatment protocol guidelines generated for the trial allowed the use of adjunctive interventions. This was inclusive of additional therapies, lifestyle advice, and nonprotocol interventions. The most commonly used additional therapies were acupressure (used on 35 of 266 patients; 13%), moxibustion (32; 12%), and electroacupuncture (19; 7%). Of the 266 patients, 176 (66%) were given some form of lifestyle advice. The most common lifestyle advice given was dietary, which was given to 113 participants (42%). This was followed by exercise advice (80/266; 30%) and relaxation advice (66/266; 25%). The most common dietary advice given was for the regulation and tonification of Spleen function, resolving of Damp and Phlegm based pathology, and tonification of Blood and Yin. Practitioners reported that they gave nonprotocol advice to 4 (2%) of the 266 patients, comprising referral to a counsellor or psychologist (*n* = 2), musculoskeletal stretches (*n* = 1), and herbal lotion application to reduce swelling and pain (*n* = 1).

After the trial, 96% of the practitioners reported applying acupuncture in exactly the same way or similarly as they did routinely. Compared to their routine practice, 91% of the practitioners reported that they gave lifestyle advice to patients “more frequently” or “about the same”. And 83% of the practitioners reported that they thought that their advice was acted upon more often or in the same way compared to their routine practice.

In terms of the two main syndrome clusters, we found some variation in acupuncture points used; see Table 5. We explored any significant associations between baseline variables and the two most common *zang fu* syndrome clusters, either associated with Liver Qi Stagnation (*n* = 108) or with Spleen Deficiency (*n* = 43). When comparing the two syndrome clusters in relation to gender using a chi-squared test, a weak trend suggested that women more commonly experienced depression with symptoms related to Spleen Deficiency whereas men's experience of depression was more likely to be related to Liver Qi Stagnation (*χ* ^2^ = 3.98, df = 1, *P* = 0.046). We found no association between the two syndrome clusters and patient's age, whether they experienced concurrent pain at baseline, baseline PHQ-9 score, whether taking antidepressant medication at the time of recruitment, or whether patients had had any thoughts that they would be better off dead or hurting themselves in some way at the outset. Using ANCOVA we found that the reported change in PHQ-9 scores between baseline and 3 months was not significantly different between the two syndrome clusters (*F* = 0.197(df2) *P* = 0.821). Both groups saw an improvement of 5 to 6 points on the PHQ-9 scale.

## 4. Discussion

Acupuncturists, when utilising the principles of traditional Chinese medicine, will routinely individualise their treatments because of the inherent variations in their patients. Treatments provided within pragmatic trials of acupuncture usually allow practitioners to vary their treatments in this regard. However, in any clinical trial, we do need a description of the intervention, in order to know what to ascribe any putative outcome to. We have utilised a method of standardisation proposed by Hawe et al. for complex interventions, in which the function and process of the intervention are standardised, thus allowing flexibility in the delivery of treatment, provided it is guided by theory [13]. In this context, we have documented the components of the intervention across 266 patients receiving acupuncture. Our report on treatments, which has been informed by the STRICTA recommendations [2], provides sufficient details for readers to interpret the results of the original trial [14] and assess the transferability of the results to other countries, settings, and populations. 

Our documentation of the syndromes diagnosed by the practitioners adds to our understanding of depression. From the perspective of traditional Chinese medicine, two syndrome clusters were commonly reported among the cohort of people in the trial with depression. The first of these clusters centres on Liver Qi Stagnation and Liver Yang Rising, for which typical symptoms along with depression might include irritability, frustration, restlessness, alternating moods, distension of the hypochondrium, and variations in muscle tension related to stress, especially around the shoulders [17]. The second cluster is related to Spleen Qi or Yang Deficiency with the possible addition of Dampness, for which typical symptoms along with depression might include tiredness, loose stools, weakness, feeling bloated after eating, a heavy sensation in the limbs, and possible loss of appetite [17]. We found the majority of patients (66%) had either a primary or a secondary diagnosis within the Liver Qi Stagnation cluster, and the second most common cluster was that of Spleen Deficiency (34%). While we did find that men were more likely to be diagnosed with the former and women with the latter, we observed no difference in three-month outcomes between the two clusters. 

In this study, we have relied on self-reports of the practitioners. Data was recorded in detailed logbooks supported by a guidance manual that set out the scope of practice, both of which were developed through a consensus process. We have assumed the accuracy of these self-reports and have no reason to doubt their veracity. In order to gain some assessment of how well the practitioners adhered to the treatment protocol, the logbooks provided the option for the practitioner to vary treatment beyond what was specified in the protocol if the variation was considered essential to the patient's treatment. These variations to the protocol were reported by the practitioners to have occurred five times. While this does in some small way compromise the integrity of the treatments provided, the fact that it happened so rarely in a cohort of 266 patients gives some support to the contention that the treatment protocol allowed practitioners to provide care that was close to their routine practice.

With regard to generalisability, we found that 96% of the practitioners in the trial reported applying acupuncture in exactly the same way or similarly compared to their routine practice outside the trial. In terms of styles of practice, 86% of British Acupuncture Council practitioners provide traditional Chinese medicine (TCM) [6], the required style for the trial. It can also be noted that the gender split of 61% female and 39% male practitioners on the trial can be compared with recent data on national percentages of British Acupuncture Council (BAcC) members (63% female and 37% male) [6]. The extent of the lifestyle advice provided within the trial, with a total of 66% of patients receiving some form of advice, can be compared to an 85% rate among BAcC practitioners [6]. Compared to their routine practice, 91% of the practitioners in the trial reported that they gave lifestyle advice to patients more frequently or in about the same way, and 83% of the practitioners reported that they thought that their advice was acted upon by patients more often or in the same way when compared to their routine practice. While there is some evidence, therefore, that the diagnostic and treatment data reported here can be generalised to acupuncturists who are members of the British Acupuncture Council practising TCM in the UK, generalising to other styles of acupuncture, especially when delivered by different professional groupings or in different countries, must be done with caution. 

## 5. Conclusion

In this paper, we have reported on 2741 treatments delivered to 266 patients receiving acupuncture from 23 practitioners. The most common syndrome cluster was that of Liver Qi Stagnation, followed by Spleen Deficiency. We found no significant differences in outcome between patients diagnosed to be within these two clusters. We found that 96% of the practitioners in the trial reported applying acupuncture in exactly the same way or similarly compared to their normal practice outside the trial. Full reporting of acupuncture treatment data, such as presented in this paper, enhances interpretation of the trial results.

## Figures and Tables

**Table 1:** Characteristics of patients, treatments, and practitioners.

Characteristic	
*Patient baseline characteristics *(*n* = 266)	
Female (%)	71
Age (mean years, median)	43, 43
Baseline PHQ-9^1^ (mean score, range)	15, 3 to 27
Baseline BDI-II^2^ (mean score, range)	31, 20 to 57
Number of previous depressive episodes (mean)	1.7
Age of first episode (mean years, median)	26, 23
Duration of depression (mean years, median)	17, 15
Experiencing concurrent pain	44%
Taking prescribed medication for depression	63%
Has had any thoughts that they would be better off dead or hurting themselves in some way (scored 1 or above on PHQ-9 question 9)	47%
*Treatment characteristics *	
Number of sessions received per patient (mean, median, range)	10.3, 12, 1 to 12
Number of weeks under treatment per patient (mean, median, range)	14, 15, 1 to 33
Approximate duration of session (mean minutes, range)	53, 28 to 95
Approximate duration needles were retained per session (mean minutes, range)	22.5, 15 to 45
*Practitioner characteristics *(*n* = 23)	
Female (number, percentage)	14 (61%)
Initial training (range in months)	36 to 48
Postqualification period (median years, range)	10, 3 to 25
Number of patients seen by each practitioner (mean, range)	12, 2 to 42
Number of treatments provided by each practitioner (mean, range)	119, 13 to 457
Number of needles used per session (mean, range)	13, 3 to 26
Needle diameter (range in mms)	0.13 to 0.35
Needle length (range in mms)	5 to 40
Average minimum depth of needle insertion (mean)	0.49 cm
Average maximum depth of needle insertion (mean)	1.41 cm
Depth of needle insertion range	0.05 to 2.5 cm
Material used (% stainless steel)	100
*Compared to your routine practice, did you provide acupuncture in the trial? *	
Exactly the same way	14 (61%)
Similarly	8 (35%)
Differently	1 (5%)
In an entirely different manner	0 (0%)
*Compared to your routine practice, did you give lifestyle advice to patients? *	
More frequently	2 (9%)
About the same	19 (82%)
Less frequently	2 (9%)
*Compared to your routine practice, to what extent do you think your advice was acted upon? *	
More often	2 (9%)
About the same	17 (74%)
Less often	4 (17%)

^1^PHQ-9 is scored from 0 to 27.

^2^BDI-II is scored from 0 to 63.

**Table 2:** Theoretical framework that guided diagnosis and treatment.

Theoretical framework	Number of patients diagnosed	Percentage of patients (total = 266)
*Zang fu* syndromes	262	99%
Fundamental substances	114	43%
8 Principles	105	39%
5 Elements	52	20%
8 Extra meridians	26	10%
Pathogenic factors	15	6%
Other	3	1%

**Table 3:** Primary *zang  fu* pattern clusters as reported by acupuncturists in 266 patients.

Primary pattern clusters	Number of patients (%)	Individual syndromes identified	Number of patients
(1) Liver Qi Stagnation	108 (41%)		
		Liver Qi Stagnation	100
		Liver Qi Stagnation and Heat	4
		Liver Invading Spleen	2
		Liver Qi Stagnation with Phlegm	1
		Liver Qi Stagnation with Yang Rising	1
(2) Spleen Deficiency	43 (16%)		
		Spleen Qi Deficiency	28
		Spleen Qi Deficiency with Dampness	11
		Spleen and Kidney Yang Deficiency with Damp	2
		Spleen Yang Deficiency	1
		Spleen and Kidney Yang Deficiency	1
(3) Heart Deficiency	20 (8%)		
		Heart Blood Deficiency	10
		Heart Yin Deficiency	8
		Heart and Liver Blood Deficiency	1
		Heart Qi Deficiency	1
(4) Kidney Deficiency	19 (7%)		
		Kidney Yang Deficiency	9
		Kidney Yin Deficiency	8
		Kidney and Liver Yin Deficiency	2
(5) Liver Deficiency	16 (6%)		
		Liver Blood Deficiency	12
		Liver and Kidney Yin Deficiency	1
		Liver and Heart Qi Deficiency	2
		Liver Blood Deficiency/Stagnation	1
(6) Heart Excess	6 (2%)		
		Heart and Liver Blood Stagnation	1
		Heart and Lung Qi Stagnation	1
		Phlegm Misting the Mind	1
		Shen Disturbance	3
(7) Other *zang fu* patterns	21 (8%)		
		Liver Yang Rising	3
		Liver Fire	5
		Heat in Pericardium	2
		Stomach Yin Deficiency	1
		Stomach Heat	2
		Lung Qi Deficiency	8
(8) Other non–*zang fu* patterns	28 (11%)		
		Yin Deficiency	3
		Blood and Yin Deficiency	7
		Damp Heat	4
		Phlegm Heat	3
		Phlegm	2
		Blood Deficiency	8
		Cold Damp in the Lower Back	1
(9)No primary pattern provided	5 (2%)		5

Total	266 (100%)		266

**Table 4:** Most commonly used acupuncture points across all patients in all sessions (*n* = 266 patients).

Rank	Acupuncture point name	No. of patients used on	No. of times point used over trial	Mean no. of times used within each course of treatment	% No. of patients used on (*n* = 266)	% times used compared to all points on the whole trial
1	Spleen 6	241	1867	7.7	91%	8.87%
2	Liver 3	237	1708	7.2	89%	8.11%
3	Stomach 36	221	1519	6.9	83%	7.21%
4	Large Intestine 4	197	1205	6.1	74%	5.72%
5	Pericardium 6	184	1075	5.8	69%	5.11%
6	Yintang	151	1072	7.1	57%	5.09%
7	Kidney 3	174	828	4.8	65%	3.93%
8	Heart 7	174	826	4.7	65%	3.92%
9	Du 20	113	654	5.8	42%	3.11%
10	Lung 7	129	586	4.5	49%	2.78%
11	Kidney 6	121	554	4.6	45%	2.63%
12	Gallbladder 34	112	490	4.4	42%	2.33%
13	Liver 8	100	450	4.5	38%	2.14%
14	Large Intestine 11	98	438	4.5	37%	2.08%
15	Spleen 9	79	369	4.7	30%	1.75%
16	Ren 12	60	339	5.7	23%	1.61%
17	Ear Shenmen/Auricular Shenmen	55	335	6.1	21%	1.59%
18	Pericardium 7	59	291	4.9	22%	1.38%
19	Kidney 7	54	241	4.5	20%	1.14%
20	Stomach 40	59	237	4.0	22%	1.13%

**Table 5:** Most commonly used acupuncture points for patients whose primary diagnosis involved one of the two main syndrome clusters.

Points used	No. of patients used on	% No. of patients used on
Liver Qi Stagnation syndrome cluster (*n* = 108)		
Liver 3	107	99.07%
Spleen 6	101	93.52%
Large Intestine 4	89	82.41%
Stomach 36	86	79.63%
Pericardium 6	82	75.93%
Yintang	71	65.74%
Heart 7	70	64.81%
Kidney 3	68	62.96%
Gallbladder 34	58	53.70%
Du 20	52	48.15%

Spleen Deficiency syndrome cluster (*n* = 43)		
Spleen 6	41	95.35%
Stomach 36	41	95.35%
Liver 3	38	88.37%
Large Intestine 4	34	79.07%
Pericardium 6	32	74.42%
Kidney 3	31	72.09%
Heart 7	28	65.12%
Du 20	25	58.14%
Yintang	25	58.14%
Spleen 9	21	48.84%

## References

[1] Manheimer E, White A, Berman B, Forys K, Ernst E (2005). Meta-analysis: acupuncture for low back pain. *Annals of Internal Medicine*.

[2] MacPherson H, Altman DG, Hammerschlag R (2010). Revised standards for reporting interventions in clinical trials of acupuncture (STRICTA): extending the CONSORT statement. *PLOS Medicine*.

[3] Prady SL, MacPherson H (2007). Assessing the utility of the standards for reporting trials of acupuncture (STRICTA): a survey of authors. *Journal of Alternative and Complementary Medicine*.

[4] Paterson C, Dieppe P (2005). Characteristic and incidental (placebo) effects in complex interventions such as acupuncture. *British Medical Journal*.

[5] Langevin HM, Wayne PM, MacPherson H (2011). Paradoxes in acupuncture research: strategies for moving forward. *Evidence-based Complementary and Alternative Medicine*.

[6] Hopton AK, Curnoe S, Kanaan M, MacPherson H (2012). Acupuncture in practice: mapping the providers, the patients and the settings in a national cross-sectional survey. *British Medical Journal Open*.

[7] Robinson N, Lorenc A, Ding W, Jia J, Bovey M, Wang X (2012). Exploring practice characteristics and research priorities of practitioners of traditional acupuncture in China and the EU—a survey. *Journal of Ethnopharmacology*.

[8] MacPherson H (2004). Pragmatic clinical trials. *Complementary Therapies in Medicine*.

[9] Beck AT, Steer RA, Brown GK (1996). *Manual for the Beck Depression Inventory-II*.

[10] MacPherson H, Richmond S, Bland MJ (2012). Acupuncture, counseling, and usual care for depression (ACUDep): study protocol for a randomized controlled trial. *Trials*.

[11] Medical Research Council (2008). *Developing and Evaluating Complex Interventions: New Guidance*.

[12] MacPherson H, Schroer S (2007). Acupuncture as a complex intervention for depression: a consensus method to develop a standardised treatment protocol for a randomised controlled trial. *Complementary Therapies in Medicine*.

[13] Hawe P, Shiell A, Riley T (2004). Complex interventions: how “out of control” can a randomised controlled trial be?. *British Medical Journal*.

[14] Schroer S, MacPherson H (2009). Acupuncture, or non-directive counselling versus usual care for the treatment of depression: a pilot study. *Trials*.

[15] Kroenke K, Spitzer RL, Williams JBW (2001). The PHQ-9: validity of a brief depression severity measure. *Journal of General Internal Medicine*.

[16] Maciocia G (1989). *The Foundations of Chinese Medicine*.

[17] Maciocia G (1994). *The Practice of Chinese Medicine: The Treatment of Diseases with Acupuncture and Chinese Herbal Medicine*.

